# Whole genome sequencing in the study of disease and application in personalised medicine

**DOI:** 10.1186/1897-4287-10-S2-A39

**Published:** 2012-04-12

**Authors:** A Kumarasuriyar

**Affiliations:** 1 Illumina, San Diego, CA, USA

## 

Next Generation Sequencing has enabled a range of applications to investigate nearly every facet of genomic science including variant detection, transcriptome profiling and epigenetic studies. Many of these applications were previously either impractical or uneconomical by Sanger sequencing. In particular, whole genome and exome sequencing are now within the reach of an increasing number of researchers due to continued reduction in costs, improvements in workflow and accessibility of appropriate technologies. Furthermore, the improvements in data handling, storage and analysis tools have contributed significantly in providing biologist-friendly methods to assemble reads and call variants.

Here I will review the recent application of whole genome and exome sequencing in both clinical and research environments for the study of breast cancer. Specifically, a recent whole-genome investigation of 50 tumour-normal pairs sourced from oestrogen receptor positive breast cancers will be explored. This study confirmed genes previously implicated in this type of cancer, as well as identifying 3 new candidates; MAP3K1, ATR and MYST. To improve clinical outcomes, it is hoped that whole genome sequencing in this study will identify treatment-resistance mechanisms for cancers sourced from women resistive to oestrogen-lowering treatment.

Additional case studies will be used to highlight how these techniques have advanced our understanding in determining the treatment and subsequent clinical outcomes for individuals suffering from rare diseases.

